# A Diagnostic Device for In-Situ Detection of Swine Viral Diseases: The SWINOSTICS Project

**DOI:** 10.3390/s19020407

**Published:** 2019-01-20

**Authors:** Concetta Montagnese, Paolo Barattini, Alessandro Giusti, Gyula Balka, Ugo Bruno, Ioannis Bossis, Athanasios Gelasakis, Matteo Bonasso, Panayiotis Philmis, Lilla Dénes, Sergio Peransi, Manuel Rodrigo, Santiago Simón, Amadeu Griol, Grzegorz Wozniakowski, Katarzyna Podgorska, Carolina Pugliese, Lapo Nannucci, Sabato D’Auria, Antonio Varriale

**Affiliations:** 1 ISS BioSense S.r.l., Via Bernardo Cavallino 113 B, 8013 Naples, Italy; concettamontagnese@gmail.com (C.M.); ugobruno595@gmail.com (U.B.); 2 Kontor 46 SaS, Via S. Francesco da Paola 6, 10123 Turin, Italy; paolo.barattini@kontor46.eu (P.B.); matteo.bonasso@kontor46.eu (M.B.); 3 CyRIC, Cyprus Research and Innovation Centre, Engomi, 2414 Nicosia, Cyprus; alessandro@cyric.eu (A.G.); info@cyric.eu (P.P.); 4 Department of Pathology, University of Veterinary Medicine, 1078 Budapest, István utca 2., Hungary balka.gyula@univet.hu (G.B.); denes.lilla@univet.hu (L.D.); 5 Department of Animal Science and Aquaculture, Agricultural University of Athens, Iera Odos 75 Str, 11855 Athens, Greece; bossisi@aua.gr (I.B.); gelasakis@aua.gr (A.G.); 6 Lumensia Sensors S.L., 46022 Valencia, Spain; speransi@lumensia.com (S.P.); mrodrigo@lumensia.com (M.R.); ssimon@lumensia.com (S.S.); 7 Nanophotonics Technology Center, Universitat Politècnica de València, 46022 Valencia Spain; agriol@upvnet.upv.es; 8 National Veterinary Research Institute, 24-100 Puławy, Poland; grzegorz.wozniakowski@piwet.pulawy.pl (G.W.); Katarzyna.Podgorska@piwet.pulawy.pl (K.P.); 9 DAGRI, University of Florence, Via delle Cascine 5, 50 144 Florence, Italy; carolina.pugliese@unifi.it (C.P.); lapo.nannucci@gmail.com (L.N.); 10 Institute of Food Science, CNR, Via Roma 64, 83100 Avellino, Italy; antonio.varriale@isa.cnr.it

**Keywords:** swine disease, photonics, antibody, ring resonator, photonic integrated circuit (PIC)

## Abstract

In this paper, we present the concept of a novel diagnostic device for on-site analyses, based on the use of advanced bio-sensing and photonics technologies to tackle emerging and endemic viruses causing swine epidemics and significant economic damage in farms. The device is currently under development in the framework of the EU Commission co-funded project. The overall concept behind the project is to develop a method for an early and fast on field detection of selected swine viruses by non-specialized personnel. The technology is able to detect pathogens in different types of biological samples, such as oral fluids, faeces, blood or nasal swabs. The device will allow for an immediate on-site threat assessment. In this work, we present the overall concept of the device, its architecture with the technical requirements, and all the used innovative technologies that contribute to the advancements of the current state of the art.

## 1. Introduction

In response to the increasing human population (estimated 9.15 billion by 2050) [1], urbanization rates, and income growth rate, the demand for livestock products during the last 40 years has dramatically increased. For example, chicken meat production has increased by a factor of nearly 5, while beef and pork production has more than doubled [2]. Confined and intensive livestock production systems in industrialized countries are the source of much of the world’s poultry and pig meat production, whereas such systems are being established in developing countries, particularly in Asia, to meet the increasing demand. The intensification of production has led to significant challenges regarding health and welfare status of farm animals which nowadays are vulnerable to various transboundary infectious agents, causing diseases such as foot-and-mouth disease, classical and African swine fever that are a continuous threat to the animal’s health and welfare and significantly undermine animals’ productivity and the sustainability of the whole value chain of meat production. 

During the last decades a reduction in the direct burden of livestock diseases has been observed, as a result of more effective treatments, vaccination and improvements in diagnostic technologies and veterinary services in developing countries [3] which are an outstanding reservoir of the emerging and re-emerging infectious diseases in livestock production. Nevertheless, swine viruses are an emerging threat already ravaging Europe. In this context, early field diagnosis is considered the first line of defense. Unluckily, to date, the time between initial disease outbreak, sample transportation and laboratory confirmation of the etiologic infectious agent can be up to several weeks or months. An early diagnosis and the establishment of reliable countermeasures to infectious disease outbreaks are essential to limit severe animal’s health and socio-economic consequences. Thus, the need for the development of mobile diagnostic units has been widely recognized. Reliable, quick and simple diagnostic testing directly on site would enable rapid evidence-based decision-making processes and targeted control strategies which are crucial to prevent further spreading of the disease. Consequently, the demand and the availability of mobile testing systems have been rapidly increasing [4,5,6,7,8].

Traditionally, virus detection in the laboratory relies on two different approaches: direct detection of the pathogen by polymerase chain reaction (PCR), enzyme-linked immunosorbent assay (ELISA) or cell culture-based virus isolation, and/or detection of circulatory antibodies in blood samples. The first approach is time consuming and need at least 2–3 h to be performed, while the later one although fast, simple and often informative, neither coincides with the timing of infection nor it necessarily correlates robustly with clinical signs, and the phase of the clinical disease.

Currently rapid diagnostics systems are designed to detect the nucleic acid or protein of the infectious agents. The vast majority of these systems rely on nucleic acid extraction, purification, and PCR-based amplification and detection. PCR amplification methods are very sensitive but often produce false positives from trace contamination of the specimen/equipment. Additionally, nucleic acid-based tests do not provide information on the viability/infectivity of the detected microorganism and often cannot be multiplexed to more than few targets.

In recent years, silicon based Photonic Integrated Circuits (PIC) have been demonstrated as a powerful platform for bio/sensing based on monoclonal antibodies. PIC can provide portable multiplex detection of proteins, with sensitivity and specificity previously unknown [9,10,11].

PICs are devices, on which different optical components and electrical parts are integrated thought the integrated optics technology and their main application is in optical fiber communications.

The method used for PICs fabrication is based on the wafer-scale technology, that including lithography technique, using as substrates silicon, silicon nitride or silica. In all cases, the employed substrate material determines features and limitations of the technology.

PICs can host large arrays of identical structures or contain complex circuit configurations. However, nowadays the achievable complexity is still not as high as for electronic integrated circuits. 

Due to these characteristics, PICs are proposed as an emerging technology that may yield novel applications in the field label-free sensor for chemical and biological detection. In fact, the tight confinement of light in integrated structures leads to an increased light-matter interaction that can be exploited to implement the detection of the analytes through the change of the optical properties of the PIC.

At the moment, the first commercial products, for different applications, were been introduced on the market (www.genalyte.com).

The SWINOSTICS (swine diseases field diagnostics toolbox) project (www.swinostics.eu) [12] aims to develop a novel field diagnostic device, based on advanced, proven, bio-sensing technologies to tackle emerging and endemic, complying with the objectives of the International Research Consortium of Animal Health (STAR-IDAZ) [13].

The SWINOSTICS approach is based on the combination of photonic integrated circuit (PIC) technology and nano-deposition technology to detect six different swine viruses.

The aim of the present paper is to describe the overall concept with particular attention to the target diseases, the state of the art in pathogen detection and the innovation of the SWINOSTICS approach. In Section 3, we present the overall design and architecture of the device and finally in Section 4, the field validation plan.

## 2. SWINOSTICS Overall Concept

The SWINOSTICS diagnostics targets are important emerging and endemic swine viruses, namely: African swine fever (ASF) [14], Classical Swine Fever (CSF) [15], Porcine Reproductive and Respiratory Syndrome (PPRS) [16], Porcine Parvovirus (PPV) [17], Porcine Circovirus 2 (PCV2) [18] and Swine Influenza A (SIV) [19]. 

The majority of existing and widely used diagnostic technologies rely on the following: (1) growing the infectious agent in cell culture [20]; (2) observing the infectious agent by light or electron microscopy [21]; (3) detecting humoral immune responses against an infectious agent with serological assays such as complement fixation, hemagglutination inhibition and ELISAs [22,23]; (4) detecting viral/microbial nucleic acid by some form of PCR/RT-PCR, hybridization with viral specific probes or non-amplification methods [24,25,26,27,28,29]; and (5) detecting viral/microbial protein antigens in tissues by immunofluorescence/immune peroxidase and in fluids by sandwich ELISAs and immune-chromatography [30,31,32,33,34,35,36]. All of them are not properly suitable for a portable filed device.

Table 1 presented the main sample media in which these viruses are detected by current diagnostic practices and by those used in our approach.

**Figure 1:**
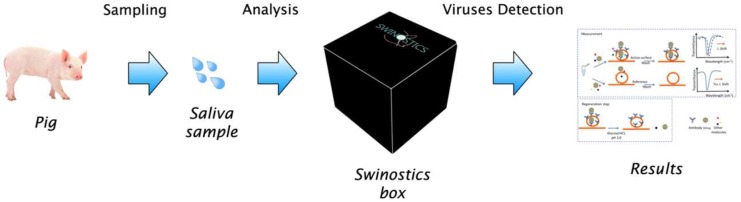
Schematic representation of the target application of the SWINOSTICS (swine diseases field diagnostics toolbox) device. Three main steps are needed: sampling, analysis and virus detection.

The SWINOSTICS device (Figure 1) will provide results in less than 30 min for five samples simultaneously, making it highly suitable for use in the field. It will be connected through a mobile application for prompt data distribution and analysis. The modular design allows future upgrades and the use in parallel of additional biosensor cartridges to increase the number of analytes simultaneously detected.

The main technical advantage is the application of three novel and already validated technologies:

1. Photonic Integrated Circuit (PIC) technology, as the basis for the development of the biosensors;
2. Label-free optical detection, based on refractive index sensing through ring resonators;
3. Nano-deposition technology [50,51], used for the biosensors. The selectivity of this technology allows the development of nano-arrays capable to simultaneously determine the presence of the different analytes of interest with high specificity.

### SWINOSTICS innovation

The SWINOSTICS innovation is at various levels. The key SWINOSTICS features are summarized below:

- The SWINOSTICS device will be the only field-use device capable of the detection of six important swine viruses of particular interest for the European and global economy.
- The device will use oral fluids as a sample for the analysis. This simplifies the sampling and minimizes the time needed for the test (no sample treatment is needed) allowing also the analysis of wild boar samples collected through adequate lures. The device will anyway be compatible with the use of other types of samples, such as faeces, blood or nasal swabs.
- The sample analysis will require less than 30 min. Additionally, five sensors, working in parallel, will be available within the device, to process up to five samples from five different animals simultaneously.
- The detection of the infectious agents is made using novel biosensors. The patented, nano-deposition technology used to develop the bio-sensing surface allows the specific determination of the presence of the biomarkers of interest.
- The PIC sensors that will be used in the device do not require the use of a fluorescent label for the detection (label-free detection).
- The increased sensitivity of the sensors is supported by the use of commercial monoclonal antibodies against the targeted viruses, which will be immobilized in an oriented manner on the PIC surface.
- The expected sensitivity and specificity levels are about 95% and 90%, respectively.
- Cost-effective and mass production suitable fabrication process of the PIC sensors. The fabrication technology will be CMOS (Complementary metal–oxide–semiconductor)-compatible.
- The device will integrate a biosensor regeneration mechanism, to make each PIC reusable for at least up to 100 times.

## 3. SWINOSTICS Device Overall Design and Architecture

**Figure 2:**
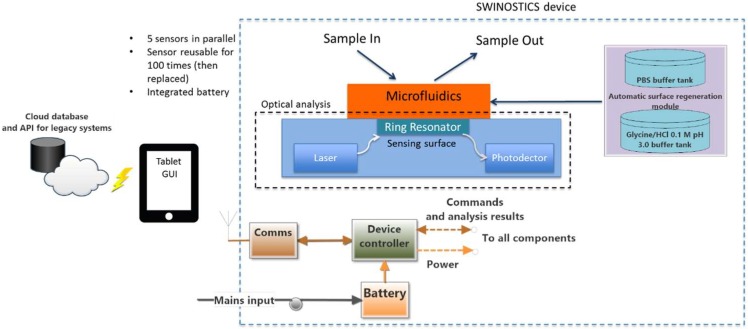
Overall design and architecture scheme. Five separate functional modules compose the SWINOSTICS device: biosensor, optical analysis module, temperature conditioning module, bio-sensing surface regeneration and preservation module and finally process, control and communication module.

In this section, we present the overall design of the SWINOSTICS device. The system requirements were identified during the initial months of the project and according to the stakeholders and the end-users’ requirements. The device is composed of five separate functional modules as shown in Figure 2: the biosensor module, the optical analysis module, the temperature conditioning module, the bio-sensing surface regeneration/preservation module and the process, control and communication module.

### 3.1. SWINOSTICS Biosensor

The analytical core module is the biosensor. It is made up of four main components: the microfluidic subsystem, the bio-sensing surface, the photonic transducer system, and the optical analysis module. These main building blocks will be controlled through the appropriate process control and communication module.

#### 3.1.1. Microfluidic Subsystem

The microfluidic subsystem is responsible for the delivery of the sample and buffer liquids to the bio-sensing surface (i.e., the inner surface to the ring resonator). It deals with the flow and distribution of fluids that are constrained to sub-millimeter channels in the dimension about 600 μm wide and 300 μm deep. The flow will be 30 μL/min. The microfluidic subsystem (chip) will be made of PMMA (Poly Methyl Methacrylate). It will consist of one single fluidic inlet and one single corresponding outlet, which is connected via tubing with a peristaltic micro-pump and allows the insertion of the bio-sensing surface sensor chip underneath (sealed by an elastomer sealing).

#### 3.1.2. Photonic Transducer

It contains the bio-sensing core. The transducer will be constructed using highly compact PIC-based detectors for biochemical sensing. CMOS technology will be used for the implementation of the PIC and in this case the underlying target design also consists of high-Q filtering structures. In each circuit there are three rings: one to compensate derivatives, another for the negative control of the detection and a third one for the measurement. 

Ring resonators are manufactured on Silicon Nitride (Si_3_N_4_) over a BOX substrate. The full layer stack is built on a silicon substrate, with a deposition of 300 nm of nitride, remaining a buried oxide layer (BOX) of 3.25 μm. PIC structures are fabricated at silicon nitride layer then an extra layer of silicon oxide is deposited as top cladding. In SWINOSTICS project, it is planned to use vertical coupling by means of focal grating couplers (GC) due to the versatility it provides to the design. Then, due to the required resolution, fabrication techniques as deep UV or electron beam are required on PIC fabrication. Each ring resonator will have its own output finished with a grating coupler. In this way, the response of each ring will be treated separately to maximize the dynamic response of the sensors and to simplify the algorithm for tracking sensor response.

PIC fabrication process will be based on standard photolithography techniques (electron beam and UV mask photolithography). A direct writing photolithography process will be optimized to transfer the patterns into a positive resist. The resist will be deposited by spin coating using an EVG101 system on the silicon nitride substrate prior to an optimized photolithography process.

**Figure 3:**
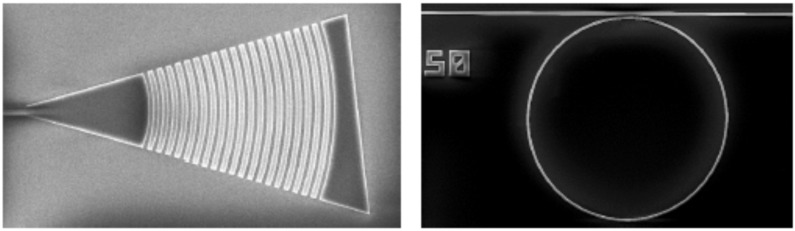
SEM images of silicon nitride grating coupler (**left**) and ring resonator (**right**).

After exposing the patterns, the resist will be developed. The developed resist could be used directly as etching mask or to create a metal mask by an evaporation/lift-off process. Finally, the silicon nitride layer will be etched by an ICP-RIE tool, which uses fluoride gases. After that, a PECVD process will be used to cover the sample/wafer with a silica upper cladding of 1 micron thick. Finally, a second photolithography step as described will be used to open windows in the sensing areas. The Figure 3 shows a SEM image of a typical grating coupler and ring resonator.

The mentioned fabrication process can be used on complete 4 or 6 inch wafers or for smaller samples, which can be employed to adjust building blocks and fabrication processes.

**Figure 4:**
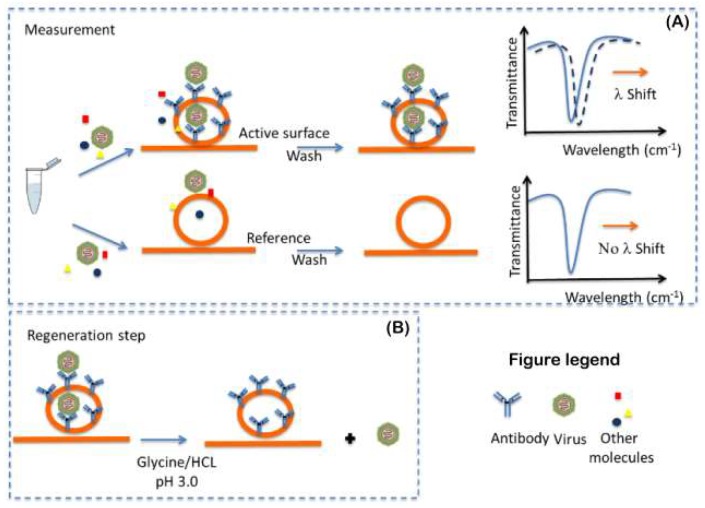
Functionalized photonic integrated circuit (PIC) surface measurement (**A**) and regeneration step (**B**).

The approach used for the functionalized PIC active-surface preparation will be to first perform derivatization with the specific reactive-group (amino and/or carboxylic group) and then functionalize it with commercial monoclonal antibodies, selected as molecular recognition elements (MREs), against the targeted viruses. As shown in Figure 4, the functionalized surface will be able to capture the specific viruses present in the sample. The antibodies will be used to derivatize the PIC surface and contribute to fundamental aspects of the sensor, such as affinity, selectivity and sensibility. For this purpose, we plan to use commercial monoclonal antibodies against the targeted viruses and immobilize in oriented manner the molecules on the surface in order to increase the performance of the surface (Figure 4) [52]. The oriented immobilization will bind the carboxyl terminal of the Fc-region to the surface to increase the antigen binding capacity of each antibody.

During the measurement, the sample will be conducted by the microfluidic system to the PIC transducer. In the transducer, if the binding of the viruses to the MRE takes place, it is translated in a positive optical signal of detection. 

The sensor system will be based on fabricating cost-effective and mass production sensors with multi-analyte capabilities consisting of an array of nano-rings. Each ring in the PIC will be functionalized with a specific probe, achieving multi-analyte sensing in a single chip. In addition, PICs will be fabricated at wafer scale with CMOS compatible technologies resulting to cost-effective sensor chips. Silicon-based nanofabrication is now a mature technology with several companies already placing silicon photonic devices on the market. Of course, there is still room for R&D in terms of more complex and higher-functionality devices. Silicon photonics is in fact being touted as the most likely successor to electronics in the interconnections between microprocessors in data centers and high-end computers. The sensors developed in SWINOSTICS will certainly benefit from both already achieved technological advances in the field and from the mass market, which will reduce the production costs due to economies of scale. To this end, the exclusive use of silicon-based materials, such as silicon nitride and silicon dioxide for the fabrication of the photonic nano-devices employed means that the fabrication technology will be CMOS-compatible. The Si_3_N_4_ waveguide has a lower refractive index contrast and shows reduced sensitivity to temperature changes with respect to Silicon on Insulator (SoI).

#### 3.1.3. Optical Analysis Module

The optical analysis module is responsible for “reading” the biosensors output. The module includes the fiber optic interfaces between the sensor cell and the optical components, such as the laser module and the detectors. The optical analysis module will operate in close conjunction with the temperature-conditioning module and with the microfluidic interface. The optical subsystem will be based on a transfer function measurement of the chip, using a tunable laser source and a photodetector connected with a fiber optic. A reference wavelength-sweeping signal from the laser will be measured continuously (power and wavelength), and the power transmitted from the circuit will be simultaneously measured.

#### 3.1.4. Temperature Conditioning Module

To provide accurate and consistent results during the analytic procedure, the developed PIC biosensors require a stable temperature. For this reason, an analysis chamber will be available within the SWINOSTICS device. A temperature of about 20 °C will be used in the analysis chamber, even though the final value will be selected after optimizing the analytic protocol. The temperature-conditioning module will be responsible for maintaining the temperature within the analysis chamber, as long as the instrument is powered. A temperature control loop will be developed, based on a temperature sensor and a Peltier actuator (for both heating and cooling) [53].

### 3.2. The Process for Bio-Sensing Surface Regeneration and Preservation

Our concept foresees the reuse of the bio-sensing surface after each measurement. In order to achieve this, post-testing, the bio-sensing surface, will be washed extensively for 5 min with a regeneration buffer to free the antibodies from the antigens. Different buffers will be tested such as glycine/HCL 50 mM pH 3.0 or ethanolamine 50 mM at pH 9.0. After this step, the surface will be washed again with a PBS buffer pH 7.4 for 10 min. Thereby the biosensor will be ready for immediate or future re-use. This will allow the reuse of the same PIC for at least up to 100 times. After this number of assays, the user will have to replace the sensor. For this purpose, a dedicated module and a specific algorithm will be developed for delivering the buffers to the PIC biosensor and controlling the regeneration procedure. Also, in order to prevent possible contamination between two measurements, due to use clinical sample, a sanitation step will be performed. For this purpose, ethanol at 70% *v*/*v* will be used to sanitize the tubes a microfluidic system before each measurement. 

### 3.3. Processing, Control and Communication Module

**Figure 5:**
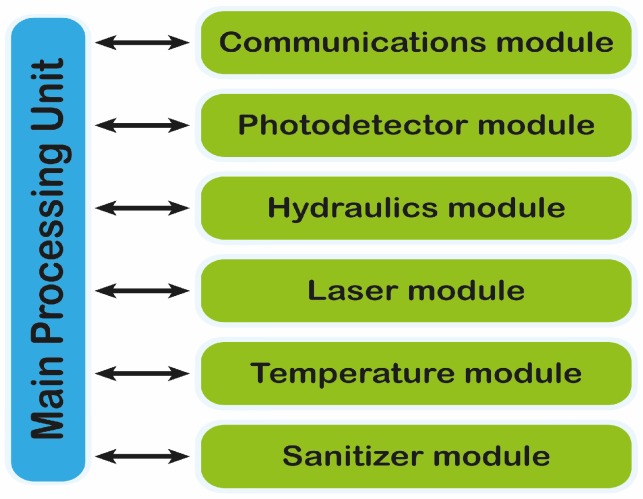
Main processing unit involved in the control and communication of the device.

The SWINOSTICS device will include its own processing unit for controlling the device operation (Figure 5), acquiring the data from the sensors, performing basic calculations and transmitting the sensor readings to a nearby tablet that will act as the user interface to the device. The processing unit also provides the necessary interfaces for communicating with the tablet (Bluetooth, Wi-Fi) and for being powered through the mains or through the integrated battery. The tablet will also communicate with a cloud-based software service where all results will be stored and immediately available to the end-users. The main device operation will be kept simple and handy requiring just the sample injection and the click of a button on the tablet interface.

## 4. Field Validation

The device, biosensors and overall approach will be validated and verified through intra-laboratory studies using both laboratory reference samples and field samples from pigs and wild boars. Validation will be done first with reference samples and then with field samples from four EU countries (Italy, Greece, Poland and Hungary) most at risk for epidemics along the geographical path of the epidemic’s diffusion. 

**Figure 6:**
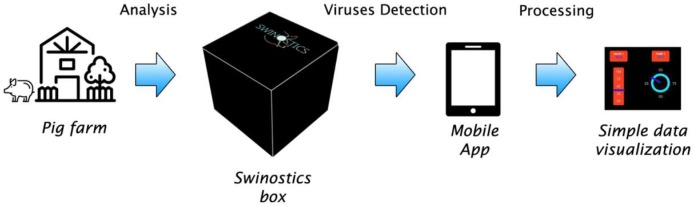
Field application of the SWINOSTICS device. The device will work on the field (Pig farm, veterinary, etc.) and the data will be transferred trough the mobile app to the cloud system.

The SWINOSTICS device will be an innovative, quicker and cheaper alternative to the current laboratory testing performed using sophisticated laboratory equipment. The proposed technical solutions will reduce the costs and the time required for testing and will make it possible to perform accurate analysis directly on the field for a broad panel of important pathogens of swine (Figure 6). 

The use of oral fluids as the main input diminishes the time needed for the analysis and simplifies the sample collection, allowing also the collection of wild boar samples through adequate licking/chewing-lures. Moreover, oral fluids are the easiest and most convenient samples to collect for farmers, veterinarians, but also for non-expert potential end-users of the device, such as hunters and the sampling includes absolutely non-invasive techniques.

Nevertheless, if the virus concentration in oral fluids proves to be too low for certain diseases, the device will be able to receive as an input pre-processed samples of different type such as nasal swabs, faeces or blood. The possibility to use different type of samples as an input is an added value, since viruses might not always be detectable in oral fluids in adequate concentration for all the targeted diseases.

## 5. Conclusions

In this paper, we describe the preliminary architecture and design of the SWINOSTICS portable device. The approach takes advantage of three novel and validated technologies: (a) Photonic Integrated Circuit (PIC) technology, (b) Label-free optical detection, based on refractive index sensing through ring resonators, (c) a nano-deposition technology used for the biosensors developing to detect in viruses in oral fluid such as saliva.

## Figures and Tables

**Table 1:** List of emerging and endemic pathogens viruses in pigs.

Targeted Virus	Sample usually Used	SWINOSTICS Approach
African Swine Fever (ASFV)	Whole blood or blood serum in live animals. Tissues from post-mortem animals such as tonsil, spleen and lymph nodes [37]	Oral fluid [38,39,40] in live animals, blood serum from post-mortem animals [41]
Porcine Reproductive and Respiratory Syndrome (PRRSV)	Whole blood and blood serum in live animals. Tissues such as lung, respiratory tract, spleen and tonsils in post-mortem animals [42])	Oral fluid and blood serum [43,44]
Swine Influenza A (SIV)	Nasal swabs and lung tissues [45]	Oral fluid and nasal swabs [46,47]
Porcine Parvovirus (PPV)	Whole mummified small foetuses/lung tissue from aborted foetuses [48,49]	Oral fluid and faeces [49]
Porcine Circovirus (PCV2)	Blood serum, bronchiolar lavage fluid, tissue homogenates	Oral fluid [18]
Classical Swine Fever (CSF)	Whole blood in live animals. Tissues such as tonsils, pharyngeal or mesenteric lymph nodes, spleen, kidney, and distal ileum in post-mortem animals [15]	Oral fluid [40,49] in live animals, blood serum from post-mortem animals [49]

## References

[1] United Nations World Population Prospects. https://population.un.org/wpp/.

[2] FAO (2010). Food and Agriculture Organization of the United Nations Statistical Databases. http://www.fao.org/.

[3] Perry B., Sones K. (2009). Global Livestock Disease Dynamics over the Last Quarter Century: Drivers, Impacts and Implications.

[4] Niemz A., Ferguson T.M. (2011). Point-of-care nucleic acid testing for infectious diseases. Trends Biotechnol..

[5] Holland C.A., Kiechle F.L. (2005). Point-of-care molecular diagnostic systems—Past, present and future. Curr. Opin. Microbiol..

[6] Park S., Zhang Y. (2011). Advances in microfluidic PCR for point-of-care infectious disease diagnostics. Biotechnol. Adv..

[7] Craw P., Balachandran W. (2012). Isothermal nucleic acid amplification technologies for point-of-care diagnostics: A critical review. Lab Chip.

[8] Asiello P.J., Baeumner A.J. (2011). Miniaturized isothermal nucleic acid amplification, a review. Lab Chip.

[9] Jahns S., Bräu M. (2015). Handheld imaging photonic crystal biosensor for multiplexed, label-free protein detection. Biomed. Opt. Express.

[10] Ksendzov A., Lin Y. (2005). Integrated optics ring-resonator sensors for protein detection. Opt. Lett..

[11] Lopez G., Estevez M. (2017). Recent advances in nanoplasmonic biosensors: Applications and lab-on-a-chip integration. Nanophotonics.

[12] Swinostics. http://swinostics.eu/.

[13] STAR—IDAZ. https://www.star-idaz.net/organisation/.

[14] ASF Regionalization in the EU. https://ec.europa.eu/food/sites/food/files/animals/docs/ad_control-measures_asf_pl-lt-regionalisation.pdf.

[15] (2009). Classical Swine Fever (Hog Cholera). http://www.oie.int/fileadmin/Home/eng/Animal_Health_in_the_World/docs/pdf/Disease_cards/CLASSICAL_SWINE_FEVER.pdf.

[16] Kuhn J.H., Lauck M. (2016). Reorganization and expansion of the nidoviral family Arteriviridae. Arch. Virol..

[17] Cui J., Biernacka K. (2017). Circulation of Porcine Parvovirus Types 1 through 6 in Serum Samples Obtained from Six Commercial Polish Pig Farms. Transbound. Emerg. Dis..

[18] Oliver-Ferrando S., Segalés J. (2016). Evaluation of natural porcine circovirus type 2 (PCV2) subclinical infection and seroconversion dynamics in piglets vaccinated at different ages. Vet. Res..

[19] Castrucci M.R., Donatelli I. (1993). Genetic reassortment between avian and human influenza A viruses in Italian pigs. Virology.

[20] Leland D.S., Ginocchio C.C. (2007). Role of Cell Culture for Virus Detection in the Age of Technology. Clin. Microbiol. Rev..

[21] Wongsrichanalai C., Barcus M.J. (2007). A review of malaria diagnostic tools: Microscopy and rapid diagnostic test (RDT). Am. J. Trop. Med. Hyg..

[22] Baron E.J., Miller J.M. (2013). A guide to utilization of the microbiology laboratory for diagnosis of infectious diseases: 2013 recommendations by the Infectious Diseases Society of America (IDSA) and the American Society for Microbiology (ASM). Clin. Infect. Dis..

[23] Reichel M.P., Lanyon S.R. (2016). Moving past serology: Diagnostic options without serum. Vet. J..

[24] Josko D. (2010). Molecular virology in the clinical laboratory. Clin. Lab. Sci..

[25] Scagnolari C., Turriziani O. (2017). Consolidation of molecular testing in clinical virology. Expert Rev. Anti-Infect. Ther..

[26] Watzinger F., Ebner K. (2006). Detection and monitoring of virus infections by real-time PCR. Mol. Asp. Med..

[27] Peccoud J., Jacob C. (1996). Theoretical uncertainty of measurements using quantitative polymerase chain reaction. Biophys. J..

[28] Broude N.E., Zhang L. (2001). Multiplex allele-specific target amplification based on PCR suppression. Proc. Natl. Acad. Sci. USA.

[29] Syvänen A.C. (2005). Toward genome-wide SNP genotyping. Nat. Genet..

[30] Lee H.H., Dineva M.A. (2010). Simple amplification-based assay: A nucleic acid-based point-of-care platform for HIV-1 testing. J. Infect. Dis..

[31] Jeong Y.J., Park K. (2006). Isothermal DNA amplification in vitro: The helicase-dependent amplification system. Cell. Mol. Life Sci..

[32] Mori Y., Notomi T. (2009). Loop-mediated isothermal amplification (LAMP): A rapid, accurate, and cost-effective diagnostic method for infectious diseases. J. Infect. Chemother..

[33] Fang X., Liu Y. (2010). Loop-mediated isothermal amplification integrated on microfluidic chips for point-of-care quantitative detection of pathogens. Anal. Chem..

[34] Yang L., Yamamoto T. (2016). Quantification of Virus Particles Using Nanopore-Based Resistive-Pulse Sensing Techniques. Front. Microbiol..

[35] Fu E., Chinowsky T. (2007). SPR imaging-based salivary diagnostics system for the detection of small molecule analytes. Ann. N. Y. Acad. Sci..

[36] Ly N., Foley K. (2007). Integrated label-free protein detection and separation in real time using confined surface plasmon resonance imaging. Anal. Chem..

[37] (2013). AFRICAN_SWINE_FEVER. https://www.oie.int/fileadmin/Home/eng/Animal_Health_in_the_World/docs/pdf/Disease_cards/AFRICAN_SWINE_FEVER.pdf.

[38] Grau F.R., Schroeder M.E. (2015). Detection of African swine fever, classical swine fever, and foot-and-mouth disease viruses in swine oral fluids by multiplex reverse transcription real-time polymerase chain reaction. J. Vet. Diagn. Investig..

[39] Davies K., Goatley L.C. (2017). Survival of African Swine Fever Virus in Excretions from Pigs Experimentally Infected with the Georgia 2007/1 Isolate. Transbound. Emerg. Dis..

[40] Petrov A., Schotte U. (2014). Alternative sampling strategies for passive classical and African swine fever surveillance in wild boar. Vet. Microbiol..

[41] Kittawornrat A., Prickett J. (2010). Porcine reproductive and respiratory syndrome virus (PRRSV) in serum and oral fluid samples from individual boars: Will oral fluid replace serum for PRRSV surveillance?. Virus Res..

[42] Pepin B.J., Kittawornrat A. (2015). Comparison of specimens for detection of porcine reproductive and respiratory syndrome virus infection in boar studs. Transbound. Emerg. Dis..

[43] Detmer S.E., Patnayak D.P. (2011). Detection of Influenza A virus in porcine oral fluid samples. J. Vet. Diagn. Investig..

[44] Decorte I., Steensels M. (2015). Detection and Isolation of Swine Influenza A Virus in Spiked Oral Fluid and Samples from Individually Housed, Experimentally Infected Pigs: Potential Role of Porcine Oral Fluid in Active Influenza A Virus Surveillance in Swine. PLoS ONE.

[45] Xiao C.T., Gerber P.F. (2012). Characterization of porcine parvovirus type 2 (PPV2) which is highly prevalent in the USA. Vet. Microbiol..

[46] Truyen U., Zimmerman J.J., Karriker L.A. (2012). Porcine Parvovirus in Diseases of Swine.

[47] Ogawa H., Taira O. (2009). Multiplex PCR and multiplex RT-PCR for inclusive detection of major swine DNA and RNA viruses in pigs with multiple infections. J. Virol. Methods.

[48] Yang Y., Qin X. (2016). Rapid and specific detection of porcine parvovirus by isothermal recombinase polymerase amplification assays. Mol. Cell. Probes.

[49] Prickett J.R., Zimmerman J.J. (2010). The development of oral fluid-based diagnostics and applications in veterinary medicine. Anim. Health Res. Rev..

[50] D’Auria S., Borini S.M., Rossi A.M., Rossi M. (2009). Process of Immobilizing Biomolecules in Porous Supports by Using an Electronic Beam. U.S. Patent.

[51] D’Auria S., Borini S.M., Rossi A.M, Rossi M. (2007). Process of Immobilizing Biomolecules in Porous Supports by Using an Electronic Beam. EP Patent.

[52] Varriale A., Bonnot K. (2016). Self-oriented monolayer immobilization of ovalbumin and B. cereus antibody molecules on a chemically modified surface of silicon nitride fosters the enhancement of capture of bio-agents. Colloids Surf. B Biointerfaces.

[53] Thermoelectric Modules. https://thermal.ferrotec.com/technology/.

